# Chronic Ileum Obstruction, Granulation, and Ulceration With IgG4-Positive Plasma Cell Infiltration in a Six-Year-Old Girl With 21trisomy

**DOI:** 10.7759/cureus.60177

**Published:** 2024-05-13

**Authors:** Hiroshi Yamanaka, Masahiro Ueki, Keisuke Kikuchi, Satoshi Yakuwa

**Affiliations:** 1 Department of Pediatrics, Obihiro Kosei Hospital, Obihiro, JPN; 2 Department of Pediatrics, Hokkaido University Hospital, Sapporo, JPN; 3 Department of Pathology, Obihiro Kosei Hospital, Obihiro, JPN

**Keywords:** fecal calprotectin, igg4-positive plasma cells, ileum obstruction, abdominal distension, 21trisomy

## Abstract

Patients with 21trisomy often develop congenital or acquired gastrointestinal diseases, such as duodenal or anal atresia, celiac disease, intussusception, and constipation. In these patients, it is often challenging to diagnose gastrointestinal diseases because most patients have difficulty explaining their complaints in detail. Furthermore, these patients also possess immunological disorders, such as increased type I interferon activation, innate immune hypersensitivity, and polarization to autoimmune. Here, we present a girl with 21trisomy and constipation who developed severe anemia, occult blood and elevated levels of calprotectin in stool, and chronic ileum obstruction confirmed by computed tomography. The patient underwent surgical resection of the ileum and recovered without complications. Pathological examination demonstrated intussusception, ischemia, ulceration, inflammatory granulation, and massive IgG4-positive plasma cell infiltration. After the surgery, her fecal calprotectin levels were normalized. We assumed that the ileum inflammation caused by ileum dilation generated ulcers and granulation, which could be associated with immunological, gastrointestinal, and intellectual disorders in patients with 21trisomy.

## Introduction

21trisomy is one of the major genetic disorders for pediatricians. Patients with 21trisomy demonstrate systemic manifestations, such as facial features including epicanthic folds, oblique palpebral fissures, a depressed nasal bridge, an upturned nose, intellectual disability, impaired motor development by low muscle tonus, congenital cardiac diseases, endocrinological and metabolic disorders, hematologic disorders, ophthalmologic diseases, and gastrointestinal diseases. Gastrointestinal diseases are one of the major complications caused by congenital (atresia of the esophagus, duodenum, ileum, or anus) or acquired reasons such as celiac diseases or constipation [1].

Abdominal distention is a common manifestation in children. The most common cause is constipation, and there are several other factors, such as obstruction by intussusception, Meckel band, inflammatory bowel disease, ascites, or functional problems such as paralytic ileus [2].

In patients with 21trisomy, severe complications of the infection and autoimmune diseases, such as thyroiditis, type I diabetes mellitus, and celiac diseases, are the characteristics. Recent studies demonstrated that these patients possess immunological disorders, such as increased type I interferon production, increased innate immune response, and polarization to autoimmunity [3-5].

Patients with 21trisomy are complicated with intellectual disabilities and could not explain their complaints in detail. Indeed, clinicians had difficulty in making the diagnosis of these patients, which could be associated with delayed diagnoses [6,7].

Here, we report a six-year-old girl with 21trisomy, ventricular septum defect, and constipation, who demonstrated severe anemia, abdominal distention, occult blood, and elevated calprotectin levels in the stool. She underwent surgical resection of the ileum and recovered without complications. Pathological examination demonstrated the intussusception with ischemia, inflammatory granulation, ulceration, and massive IgG4-positive plasma cell infiltration around the ulcer. These manifestations were caused by the intellectual, gastrointestinal, and immunological disorders in the patient with 21trisomy.

## Case presentation

The patient developed mild respiratory distress at birth, although there were no significant signs during pregnancy. She was diagnosed with transient tachypnea of the newborn and ventricular septum defect. Also, distinct facial characteristics such as a flat face and upward palpebral slant were noticed. G-band karyotyping test demonstrated 21 trisomy. She received regular evaluations from the pediatricians. She developed constipation treated with high dose picosulfate sodium and magnesium oxide at 1.5 years old and moderate developmental retardation.

**Figure 1:**
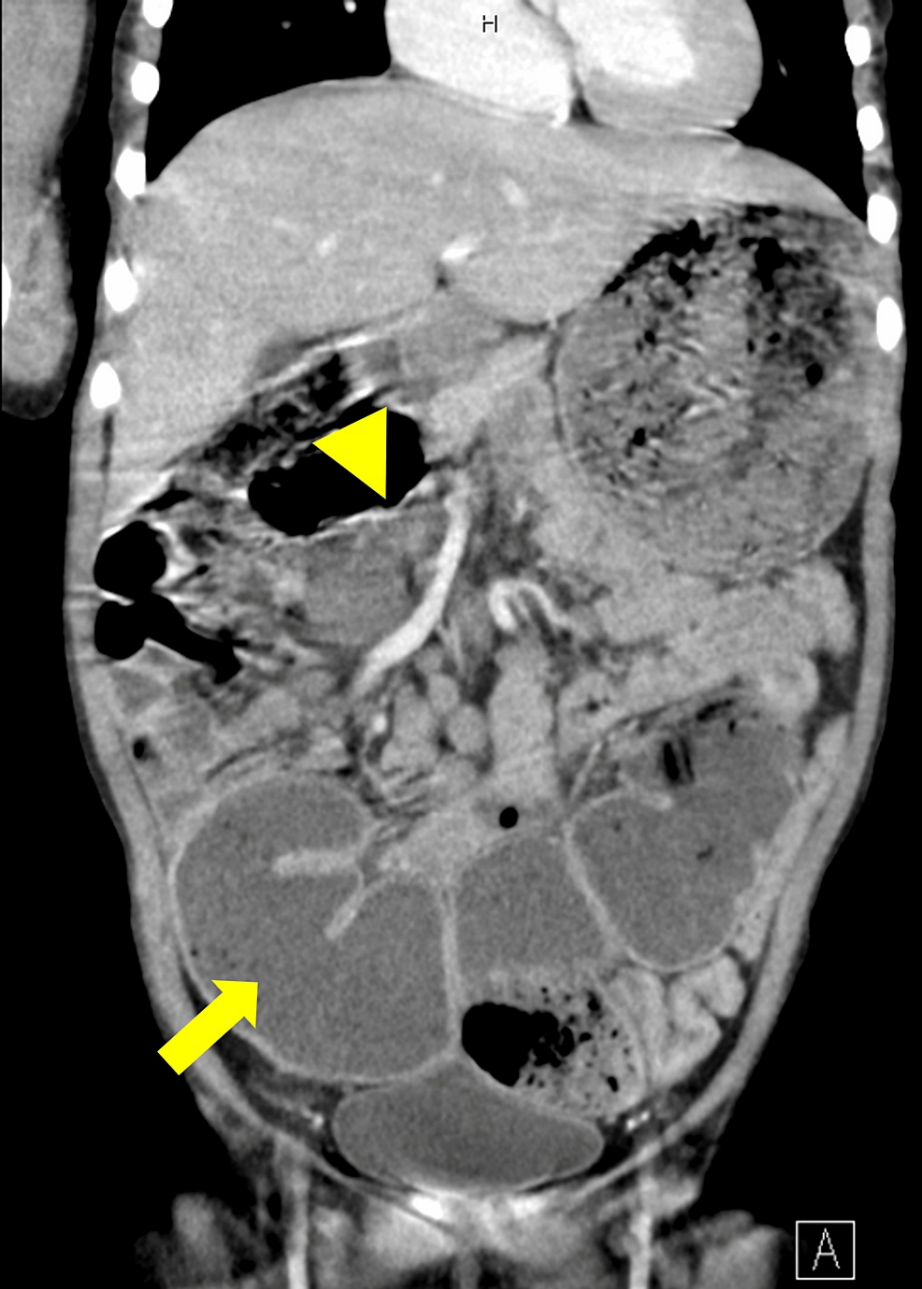
Dilated ileum (indicated with an arrow) and possible stenosis (indicated with an arrowhead) in a contrast-enhanced computed tomography scan.

At six years old, she came to the hospital for a regular assessment and demonstrated lethargy and a pale face, although previous laboratory examinations performed nine months ago showed no anemia (Hb 14.0 g/dl). Although the patient did not show significantly decreased oral intake or vomit, physical examination demonstrated abdominal distention and mild tenderness of the upper abdominal area without muscular defense. Laboratory examination data was as follows: WBC 6200 cells/μl (neut 64%, lymph 28%, mono 6.0%, Eos 1.0%), RBC 2.65x10^6 cells/μl, Hb 4.3 g/dl, hematocrit 18.0%, mean corpuscular volume (MCV) 67.9 fl, mean corpuscular hemoglobin (MCH) 16.2 pg, mean corpuscular hemoglobin concentration (MCHC) 23.9%, platelet (Plt) 476 x10^3/μl. Prothrombin time-international normalized ratio (PT-INR) 0.98, activated partial thromboplastin time (APTT) 23.8 sec, blood urea nitrogen (BUN) 19.3 mg/dl, Cre 0.38 mg/dl, Fe 0.7 μg/L, unsaturated iron-binding capacity (UIBC) 44.3 μg/L, ferritin 5.0 ng/ml, C-reactive protein 0.6 mg/L, IgG 9.61 g/L, IgG4 0.823 g/L, IgA 1.73 g/L, IgM 0.56 g/L. Severe microcytic hypochromic anemia was confirmed, whereas no overt bleeding was noticed in this period. A detailed examination demonstrated occult blood (Hb 551 U/L) and elevated calprotectin levels (2650 μg/g, normal range < 50 μg/g) in the stool. An enlarged ileum with possible stenosis was confirmed in the contrast-enhanced computed tomography (CT) scan (Figure 1). Since there was no sign of ischemia, she was treated conservatively with fasting and iron supplements. The patient got well after a resolution of anemia and took foods and drinks orally without vomiting, although abdominal distention was not resolved.

**Figure 2:**
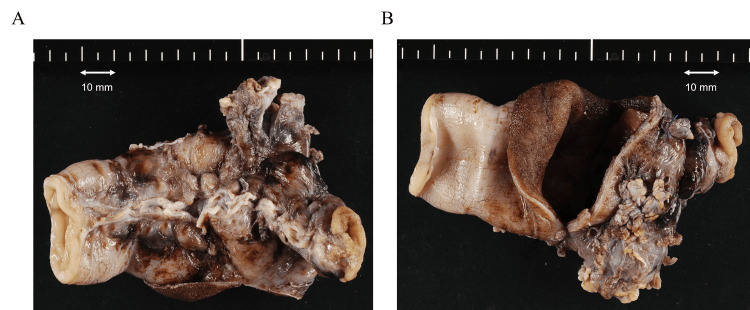
Focal dilation and ischemic change in the resected ileum. The oral side is located on the left of the photograph. An Outer appearance without section B Internal appearance of focal dilation with section.

After two months, she received the contrast-enhanced CT scan which demonstrated persistent ileum dilation. She underwent exploratory laparoscopic surgery for possible stenosis or ileum duplex. Pediatric surgeons found stenosis in the middle section of the ileum and resected it. The resected intestine showed short intussusception (Figure 2).

**Figure 3:**
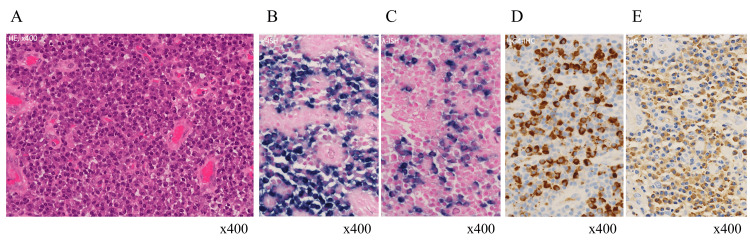
Massive filtration of IgG4-positive plasma cells in the ileum by pathological examination. A: Massive plasma cell infiltration in hematoxylin-eosin staining; B and C: Normal surface immunoglobulin by in-situ hybridization. κ-chain (B) and λ-chain (C); D and E: Significant IgG4 positive cells by immunohistochemistry of IgG4 (D) and IgG (E).

Pathological examination demonstrated incarceration, ulcer, granulation, and massive infiltration of inflammatory cells, predominantly IgG4-positive plasma cells (Figure 3). Infiltration of IgG4-positive cells was found only around the ulcer and granulation. After the surgery, the ileum dilation did not relapse and the fecal calprotectin level was normalized. She got well without complications.

## Discussion

Our patient demonstrated chronic obstruction by intussusception. Although ischemia was confirmed by pathological examination, severe necrosis or perforation was not noted. Intussusception except in infants could be associated with organic problems such as lymphoma or Meckel’s diverticulum. In this patient, granulation and ulceration with IgG4-positive plasma cell infiltration was the only manifestation that could be associated with intussusception.

Related to the intestinal inflammation, elevated levels of fecal calprotectin were confirmed in this patient. Fecal calprotectin is one of the neutrophilic intestinal inflammation markers. Its elevation was reported in inflammatory bowel diseases, granulomatous colitis in chronic granulomatous disease, celiac disease, and food-protein-induced enterocolitis [8,9]. As her symptoms and laboratory data were not compatible with these diseases, we assumed that neutrophilic inflammation and granulation were associated with elevated fecal calprotectin levels. In addition, neutrophils and monocytes in patients with 21trisomy demonstrate hypersensitivity to lipopolysaccharide [3]. Intestinal mucosal damage by chronic constipation and ileum dilation could permit enterobacterial invasion leading to hyperinflammation by neutrophils and monocytes. Indeed, elevated levels of calprotectin were normalized after the surgery (41 ug/g).

Associated with inflammation, massive IgG4-positive plasma cell infiltration and mild fibrosis were confirmed in our patient. Although these manifestations were one of the characteristics of IgG4-related diseases, granulation was one of the exclusion criteria for IgG4-related diseases [10]. Furthermore, IgG4-positive plasma cell infiltration was confirmed only around the granulation and ulcer in this patient. IgG4-positive plasma cell infiltration is also found in inflammatory bowel disease [11]. In patients with inflammatory bowel diseases who underwent surgical resection of the intestine or endoscopic biopsy, approximately 20% to 40% of patients demonstrated IgG4-positive plasma cell infiltration [12,13]. Although the pathophysiology of IgG4-positive plasma cell infiltration has not been determined, this phenomenon could be associated with the activation or suppression of immune cells in the intestine. We assumed that a similar immunological response occurred in the patient’s ileum.

Although the patient does not demonstrate any symptoms associated with irritable bowel disease or IgG4-related diseases after the surgery, successive evaluation of the development of these diseases is necessary.

## Conclusions

We experienced a patient with 21trisomy developing severe anemia and ileum stenosis. This case is rare, but it could suggest the importance of careful evaluation of abdominal distention including the treatment of the constipation, especially in patients with 21trisomy. To evaluate abnormal intestinal conditions by the inflammation, fecal calprotectin could be a potential marker, although more detailed evaluations are needed.

## References

[1] Ravel A, Mircher C, Rebillat AS, Cieuta-Walti C, Megarbane A (2020). Feeding problems and gastrointestinal diseases in Down syndrome. Arch Pediatr.

[2] Forootan M, Bagheri N, Darvishi M (2018). Chronic constipation: a review of literature. Medicine (Baltimore).

[3] Huggard D, McGrane F, Lagan N (2018). Altered endotoxin responsiveness in healthy children with Down syndrome. BMC Immunol.

[4] Malle L, Martin-Fernandez M, Buta S, Richardson A, Bush D, Bogunovic D (2022). Excessive negative regulation of type I interferon disrupts viral control in individuals with Down syndrome. Immunity.

[5] Malle L, Patel RS, Martin-Fernandez M (2023). Autoimmunity in Down's syndrome via cytokines, CD4 T cells and CD11c(+) B cells. Nature.

[6] Alshaibani F (2022). Superior mesenteric artery syndrome in Down syndrome: a case report. Cureus.

[7] Al-Nattah M, Abdullah A, Alkhateeb N, Abu Qaoud H, Al Ali A, Alzakeebeh O (2023). Navigating a complex presentation: management of hypernatremic dehydration, acute kidney injury, hyperkalemia, and metabolic acidosis in a patient with Down syndrome: a case report. Cureus.

[8] Asiri AS, Algarni SS, Althubaiti AQ, Alzubaidi MA, Alghamdi JA, Almalki GA (2023). Fecal calprotectin and organic gastrointestinal disease: a systematic review. Cureus.

[9] Zain-Alabedeen S, Kamel N, Amin M, Vernon-Roberts A, Day AS, Khashana A (2023). Fecal calprotectin and cow’s milk-related-symptoms score in children with cow’s milk protein allergy. Pediatr Gastroenterol Hepatol Nutr.

[10] Umehara H, Okazaki K, Kawa S (2021). The 2020 revised comprehensive diagnostic (RCD) criteria for IgG4-RD. Mod Rheumatol.

[11] Chen X, Sun W, Lin R, Huang Z, Chen W (2018). IgG4+ plasma cell infiltration is correlated with the development of inflammatory bowel disease and can be regulated by TLR-4. Int J Clin Exp Pathol.

[12] Topal F, Sarıtaş Yüksel E, Ekinci N, Pekdiker M, Cakalağaoğlu F, Alper E, Unsal B (2014). The prevalence of IgG4-positive plasma cell infiltrates in inflammatory bowel disease patients without autoimmune pancreatitis. Turk J Gastroenterol.

[13] Wang Z, Zhu M, Luo C (2018). High level of IgG4 as a biomarker for a new subset of inflammatory bowel disease. Sci Rep.

